# Use of 13-Valent Pneumococcal Conjugate Vaccine and 23-Valent Pneumococcal Polysaccharide Vaccine Among Adults Aged ≥65 Years: Updated Recommendations of the Advisory Committee on Immunization Practices

**DOI:** 10.15585/mmwr.mm6846a5

**Published:** 2019-11-22

**Authors:** Almea Matanock, Grace Lee, Ryan Gierke, Miwako Kobayashi, Andrew Leidner, Tamara Pilishvili

**Affiliations:** ^1^National Center for Immunization and Respiratory Diseases, CDC; ^2^Stanford University, Stanford, California.

## Introduction

Two pneumococcal vaccines are currently licensed for use in adults in the United States: a 13-valent pneumococcal conjugate vaccine (PCV13 [Prevnar 13, Pfizer, Inc.]) and a 23-valent pneumococcal polysaccharide vaccine (PPSV23 [Pneumovax 23, Merck and Co., Inc.]). In 2014, the Advisory Committee on Immunization Practices (ACIP)* recommended routine use of PCV13 in series with PPSV23 for all adults aged ≥65 years based on demonstrated PCV13 safety and efficacy against PCV13-type pneumonia among adults aged ≥65 years (*1*). At that time, ACIP recognized that there would be a need to reevaluate this recommendation because it was anticipated that PCV13 use in children would continue to reduce disease burden among adults through reduced carriage and transmission of vaccine serotypes from vaccinated children (i.e., PCV13 indirect effects). On June 26, 2019, after having reviewed the evidence accrued during the preceding 3 years (https://www.cdc.gov/vaccines/acip/recs/grade/PCV13.html), ACIP voted to remove the recommendation for routine PCV13 use among adults aged ≥65 years and to recommend administration of PCV13 based on shared clinical decision-making for adults aged ≥65 years who do not have an immunocompromising condition,^†^ cerebrospinal fluid (CSF) leak, or cochlear implant, and who have not previously received PCV13. ACIP recognized that some adults aged ≥65 years are potentially at increased risk for exposure to PCV13 serotypes, such as persons residing in nursing homes or other long-term care facilities and persons residing in settings with low pediatric PCV13 uptake or traveling to settings with no pediatric PCV13 program, and might attain higher than average benefit from PCV13 vaccination. When patients and vaccine providers^§^ engage in shared clinical decision-making for PCV13 use to determine whether PCV13 is right for a particular person, considerations might include both the person’s risk for exposure to PCV13 serotypes and their risk for developing pneumococcal disease as a result of underlying medical conditions. All adults aged ≥65 years should continue to receive 1 dose of PPSV23. If the decision is made to administer PCV13, it should be given at least 1 year before PPSV23. ACIP continues to recommend PCV13 in series with PPSV23 for adults aged ≥19 years with an immunocompromising condition, CSF leak, or cochlear implant (*2*).

## Background

*Streptococcus pneumoniae* (pneumococcus) can cause serious illness, including sepsis, meningitis, and pneumonia with bacteremia (invasive) or without bacteremia (noninvasive). Since the early 1980s, PPSV23 has been recommended for persons aged ≥2 years with certain underlying medical conditions, and all adults aged ≥65 years (*3*). 7-valent pneumococcal conjugate vaccine (PCV7) was introduced into the routine pediatric immunization schedule in 2000 and was replaced by PCV13 in 2010 (*4*). In 2012, PCV13 was recommended in series with PPSV23 for adults aged ≥19 years with immunocompromising conditions, CSF leaks, or cochlear implants (*2*). In 2014, PCV13 was recommended for all adults aged ≥65 years (*1*,*5*). Widespread use of PCV7 and PCV13 in children has led to sharp declines in pneumococcal disease among unvaccinated children and adults by preventing carriage, and thereby transmission, of vaccine-type strains (Figure). In 2014, ACIP recognized that, while in the short-term, routine PCV13 use among adults aged ≥65 years was warranted, in the long-term, continued indirect effects from PCV13 use in children might limit the utility of this recommendation. In addition, models predicted limited public health benefits in the long-term, given the relatively low remaining PCV13-type disease burden (*1*). Therefore, ACIP proposed that the recommendation for routine PCV13 use among adults aged ≥65 years be evaluated 4 years after implementation of the 2014 recommendation.

**FIGURE:**
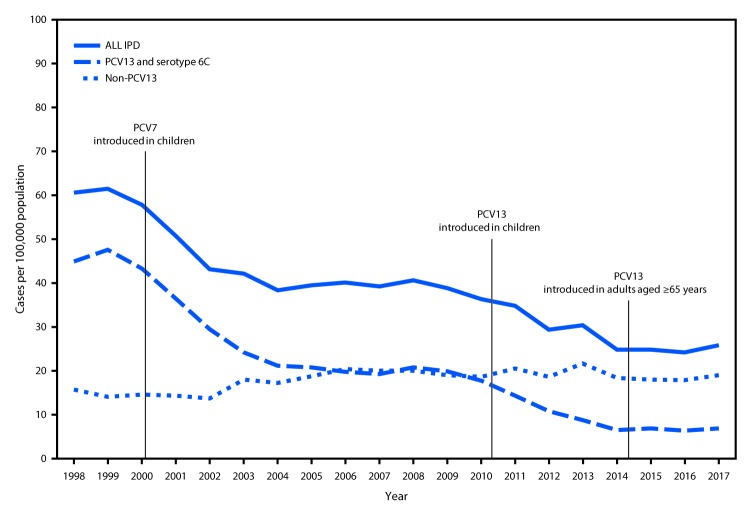
Invasive pneumococcal disease (IPD) incidence among adults aged ≥65 years, by pneumococcal serotype* — United States, 1998–2017 **Source**: Active Bacterial Core Surveillance, unpublished data, 2019. **Abbreviations**: PCV = pneumococcal conjugate vaccine; PCV7 = 7-valent PCV (serotypes 4, 6B, 9V, 14, 18C, 19F, and 23F); PCV13 = 13 valent PCV (PCV7 serotypes plus 1, 3, 5, 6A, 19A and 7F). * Serotype 6C showed cross-protection from 6A antigen in PCV13 and was grouped with PCV13 serotypes for IPD.

## Methods

During 2016–2019, using the Evidence to Recommendations Framework, (https://www.cdc.gov/vaccines/acip/recs/grade/PCV13-etr.html) the ACIP Pneumococcal Vaccines Work Group reviewed relevant scientific evidence regarding the benefits and harms of PCV13 use among adults aged ≥65 years without an immunocompromising condition, CSF leak, or cochlear implant, in the context of >5 years of pediatric PCV13 use. The Work Group evaluated the quality of evidence for PCV13 efficacy, effectiveness, safety, and population-level impact on pneumococcal-related disease using the Grading of Recommendations Assessment, Development and Evaluation (GRADE) approach (https://www.cdc.gov/vaccines/acip/recs/grade/PCV13.html).

A systematic review of scientific literature published from January 1, 2014, to July 3, 2018, was conducted to identify studies evaluating direct and indirect effects of vaccination with PCV13 on invasive pneumococcal disease (IPD), pneumonia (PCV13-type,^¶^ all pneumococcal, and all-cause), and mortality (pneumococcal or all-cause). In addition, PCV13 safety was evaluated by looking for severe adverse events, including death, occurring after receipt of PCV13 in adults aged ≥65 years. Title and abstract screening yielded 364 studies for in-depth review. Of these, 344 did not use PCV13 or did not include an outcome or population of interest. Observational studies with <20% adult PCV13 coverage and studies conducted in settings with low pediatric PCV13 coverage were excluded, as were studies evaluating PCV13 safety if PCV13 was administered with another vaccine, because severe adverse events could not be attributed to PCV13. The remaining 20 studies were included in the GRADE tables. The policy question considered was whether PCV13 should be administered routinely to all immunocompetent** adults aged ≥65 years in the context of indirect effects from pediatric PCV use experienced to date.

## Summary of Evidence

**PCV13 effectiveness and safety (individual-level benefits and harms).** Before the 2014 recommendation, a randomized placebo-controlled Community-Acquired Pneumonia Immunization Trial in Adults (CAPiTA) conducted in the Netherlands demonstrated 75% (95% confidence interval [CI] = 41%–91%) efficacy against PCV13-type IPD and 45% (CI = 14%–65%) efficacy against noninvasive PCV13-type pneumonia among adults aged ≥65 years (*6*). Postlicensure studies included in the GRADE tables in 2019 (https://www.cdc.gov/vaccines/acip/recs/grade/PCV13.html) demonstrated PCV13 effectiveness against PCV13-type IPD (47%–59%) (*7*,*8*), noninvasive PCV13-type pneumonia (38%–70%) (*9*,*10*), and all-cause pneumonia (6%–11%) (*11*,*12*). PCV13 efficacy was not demonstrated against PCV13-type or all-cause mortality (*6*); no studies evaluating PCV13 effectiveness against mortality were identified. Three randomized controlled trials (*6*,*13*,*14*) and six observational studies (*15*–*20*) that assessed harms were evaluated (https://www.cdc.gov/vaccines/acip/recs/grade/PCV13.html). The rates of severe adverse events were similar among participants vaccinated with PCV13 versus placebo or PPSV23 (https://www.cdc.gov/vaccines/acip/recs/grade/PCV13.html). Common reported PCV13-associated adverse reactions included pain, redness, and swelling at the injection site, limitation of movement of the arm in which the injection was given, fatigue, headache, chills, decreased appetite, generalized muscle pain, and joint pain (*21*). Overall, PCV13 was assessed to be safe and effective in preventing PCV13-type IPD and noninvasive pneumonia.

**PCV13 population-level impact (indirect and direct effects) on disease among adults aged ≥65 years.** The U.S. pediatric PCV program has been successful in preventing disease among young children through direct protection of vaccinated children as well as in unvaccinated populations through indirect effects (Figure). The incidence of PCV13-type IPD among adults aged ≥65 years declined ninefold during 2000–2014, before the adult PCV13 program was implemented (*22*). During the same period, indirect effects of similar magnitude were observed among adults aged ≥65 years at increased risk for IPD because of either older age (≥85 years) (*22*,*23*) or presence of underlying chronic medical conditions (*24*). Indirect effects on PCV13-type and all-cause pneumonia among adults have also been demonstrated since 2000 (*25*–*27*). In 2014, additional reductions in disease incidence among adults aged ≥65 years were expected to occur as a result of ongoing indirect effects of the pediatric PCV13 program, as well as through direct effects of PCV13 use among adults. PCV13 uptake among adults aged ≥65 years increased rapidly, with coverage in 2018 estimated at 47%; coverage with any pneumococcal vaccine was 62%, with PPSV23 was 45%, and with both PCV13 and PPSV23 was 30% (*23*). However, from 2014–2017, no further reduction in PCV13-type IPD incidence was observed among adults aged ≥65 years, with the incidence stable at five of 100,000 population (20% of all IPD) (*22*). Similarly, since 2014, no impact on PCV13-type IPD incidence has been observed among adults aged 19–64 years, a population only experiencing indirect PCV13 effects during this period. During 2014–2016, no reduction in the incidence of noninvasive pneumococcal pneumonia (all serotypes combined) was observed among adults (*28*). One recent unpublished cohort study found a 31.5% reduction in PCV13-type pneumonia and a 13.8% reduction in all-cause pneumonia between 2014–2015 and 2015–2016 (*29*). In this study, PCV13-types contributed to 4% of all-cause pneumonia among adults aged ≥65 years during 2015–2016 (*29*) compared with the estimated 10% in 2014 (*1*). Overall, since the 2014 recommendation for PCV13 use among adults, minimal changes in the incidence of pneumococcal disease among adults at the population-level were observed, through both direct PCV13 effects from vaccinating older adults and continued indirect effects from PCV13 use in children.

**Economic analyses.** Two independent economic models evaluated the expected public health impact and cost effectiveness of continued PCV13 use in series with PPSV23 versus use of PPSV23 alone. These models estimated that, over the lifetime of a single cohort of 2.7 million adults aged 65 years, an expected 76–175 cases of PCV13-type IPD and 4,000–11,000 cases of PCV13-type pneumonia would be averted through continued PCV13 use in series with PPSV23, compared with PPSV23 alone (*30*). Applying the total costs to quality adjusted life years (QALY), the estimated cost effectiveness ratios were $200,000 to $560,000 per QALY. In 2014, the estimated cost per QALY for PCV13 use in series with PPSV23 was $65,000 (*31*). Considering the range of values for sensitivity analyses for key inputs in these models, the results of the economic analyses were less favorable toward continued PCV13 use for all adults aged ≥65 years compared with PPSV23 alone.

## Rationale

Incidence of PCV13-type disease has been reduced to historically low levels among adults aged ≥65 years through indirect effects from pediatric PCV13 use. Implementation of a PCV13 recommendation for all adults aged ≥65 years in 2014 has had minimal impact on PCV13-type disease at the population level in this age group. However, PCV13 is a safe and effective vaccine that can reduce the risk for PCV13-type IPD and noninvasive pneumonia among persons aged ≥65 years. Balancing this evidence and considering acceptability and feasibility concerns, in June 2019 ACIP voted to no longer routinely recommend PCV13 for all adults aged ≥65 years and instead, to recommend PCV13 based on shared clinical decision-making for adults aged ≥65 years who do not have an immunocompromising condition, CSF leak, or cochlear implant (Table 1) (Table 2).

**TABLE 1:** Recommendations for 13-valent pneumococcal conjugate vaccine (PCV13) and 23-valent pneumococcal polysaccharide vaccine (PPSV23) among adults aged ≥19 years

Medical indication group	Specific underlying medical condition	PCV13 for persons aged ≥19 years	PPSV23* for persons aged 19–64 years	PCV13 for persons aged ≥65 years	PPSV23 for persons aged ≥65 years
None	None of the below	No recommendation	No recommendation	Based on shared clinical decision-making^†^	1 dose; if PCV13 has been given, then give PPSV23 ≥1 year after PCV13
Immunocompetent persons	Alcoholism	No recommendation	1 dose	Based on shared clinical decision-making^†^	1 dose; if PCV13 has been given, then give PPSV23 ≥1 year after PCV13 and ≥5 years after any PPSV23 at age <65 years
	Chronic heart disease^§^				
	Chronic liver disease				
	Chronic lung disease^¶^				
	Cigarette smoking				
	Diabetes mellitus				
	Cochlear implant	1 dose	1 dose ≥8 weeks after PCV13	1 dose if no previous PCV13 vaccination	1 dose ≥8 weeks after PCV13 and ≥5 years after any PPSV23 at <65 years
	CSF leak				
Immunocompromised persons	Congenital or acquired asplenia	1 dose	2 doses, 1st dose ≥8 weeks after PCV13 and 2nd dose ≥5 years after first PPSV23 dose	1 dose if no previous PCV13 vaccination	1 dose ≥8 weeks after PCV13 and ≥5 years after any PPSV23 at <65 years
	Sickle cell disease/other hemoglobinopathies				
	Chronic renal failure				
	Congenital or acquired immunodeficiencies**				
	Generalized malignancy				
	HIV infection				
	Hodgkin disease				
	Iatrogenic immunosuppression^††^				
	Leukemia				
	Lymphoma				
	Multiple myeloma				
	Nephrotic syndrome				
	Solid organ transplant				

**Abbreviations**: CSF = cerebrospinal fluid; HIV = human immunodeficiency virus.

* Only refers to adults aged 19–64 years. All adults aged ≥65 years should receive 1 dose of PPSV23 ≥5 years after any previous PPSV23 dose, regardless of previous history of vaccination with pneumococcal vaccine. No additional doses of PPSV23 should be administered following the dose administered at age ≥65 years.

^†^ Recommendations that changed in 2019.

^§^ Includes congestive heart failure and cardiomyopathies.

^¶^ Includes chronic obstructive pulmonary disease, emphysema, and asthma.

** Includes B- (humoral) or T-lymphocyte deficiency, complement deficiencies (particularly C1, C2, C3, and C4 deficiencies), and phagocytic disorders (excluding chronic granulomatous disease).

^††^ Diseases requiring treatment with immunosuppressive drugs, including long-term systemic corticosteroids and radiation therapy.

**TABLE 2:** Policy options* for use of pneumococcal vaccines in adults aged ≥65 years presented for a vote and considerations by the Advisory Committee on Immunization Practices (ACIP), June 2019

Proposed policy	Considerations raised at the June 2019 ACIP meeting		Outcome (votes in favor: against)
	In favor	Against	
ACIP recommends PCV13 for all adults aged ≥65 years who have not previously received PCV13. PCV13 should be given first, followed by a dose of PPSV23	PCV13 is effective against invasive pneumococcal disease and pneumonia	Low burden of PCV13-type disease remaining	Rejected (6:8)
	Changing the recommendation could negatively impact the perceived importance of adult pneumococcal vaccine recommendations	Population-level impact from PCV13 use among older adults observed to date has been minimal	
	Universal recommendations are easier for clinicians to understand and implement than the recommendation based on shared clinical decision-making	Universal PCV13 recommendation for older adults are not a judicious use of resources	
ACIP no longer recommends PCV13 for adults aged ≥65 years who do not have an immunocompromising condition,^†^ CSF leak, or cochlear implant. All adults aged ≥65 years should receive a dose of PPSV23	Largest public health benefit for older adults is gained through indirect effects from pediatric PCV13 use	PCV13 is effective against PCV13-type invasive pneumococcal disease and pneumonia	Rejected (1:13)
ACIP recommends PCV13 based on shared clinical decision-making for adults aged ≥65 years who do not have an immunocompromising condition,^†^ CSF leak, or cochlear implant and who have not previously received PCV13.All adults aged ≥65 years should receive a dose of PPSV23	Balances the minimal population-level impact of a routine recommendation with the potential for individual-level protection	—^§^	Affirmed (13:1)
	PCV13 would remain available to patients who want this added protection		

**Abbreviations:** CSF = cerebrospinal fluid; PCV13 = 13-valent pneumococcal conjugate vaccine; PPSV23 = 23-valent pneumococcal polysaccharide vaccine.

* Policy options listed in the order they were presented to ACIP for a vote.

^†^ Includes adults with chronic renal failure, nephrotic syndrome, immunodeficiency, iatrogenic immunosuppression, generalized malignancy, human immunodeficiency virus, Hodgkin disease, leukemia, lymphoma, multiple myeloma, solid organ transplants, congenital or acquired asplenia, sickle cell disease, or other hemoglobinopathies.

^§^ No content for this cell.

## New Pneumococcal Vaccine Recommendations for Adults Aged ≥65 Years Old

**PCV13.** PCV13 vaccination is no longer routinely recommended for all adults aged ≥65 years. Instead, shared clinical decision-making for PCV13 use is recommended for persons aged ≥65 years who do not have an immunocompromising condition, CSF leak, or cochlear implant and who have not previously received PCV13 (Table 1).

**CDC guidance for shared clinical decision-making.** When patients and vaccine providers engage in shared clinical decision-making for PCV13 use to determine whether PCV13 is right for the specific individual aged ≥65 years, considerations may include the individual patient’s risk for exposure to PCV13 serotypes and the risk for pneumococcal disease for that person as a result of underlying medical conditions (Box).

BOX **Considerations for shared clinical decision-making regarding use of 13-valent pneumococcal conjugate vaccine (PCV13) in adults aged ≥65 years**

- PCV13 is a safe and effective vaccine for older adults. The risk for PCV13-type disease among adults aged ≥65 years is much lower than it was before the pediatric program was implemented, as a result of indirect PCV13 effects (by preventing carriage and, thereby, transmission of PCV13-type strains). The remaining risk is a function of each individual patient’s risk for exposure to PCV13 serotypes and the influence of underlying medical conditions on the patient’s risk for developing pneumococcal disease if exposure occurs.
- The following adults aged ≥65 years are potentially at increased risk for exposure to PCV13 serotypes and might attain higher than average benefit from PCV13 vaccination, and providers/practices caring for many patients in these groups may consider regularly offering PCV13 to their patients aged ≥65 years who have not previously received PCV13:
  - Persons residing in nursing homes or other long-term care facilities
  - Persons residing in settings with low pediatric PCV13 uptake
  - Persons traveling to settings with no pediatric PCV13 program
- Incidence of PCV13-type invasive pneumococcal disease and pneumonia increases with increasing age and is higher among persons with chronic heart, lung, or liver disease, diabetes, or alcoholism, and those who smoke cigarettes or who have more than one chronic medical condition.* Although indirect effects from pediatric PCV13 use were documented for these groups of adults and were comparable to those observed among healthy adults, the residual PCV13-type disease burden remains higher in these groups. Providers/practices caring for patients with these medical conditions may consider offering PCV13 to such patients who are aged ≥65 years and who have not previously received PCV13.

* Ahmed SS, Pondo T, Xing W, et al. Early impact of 13-valent pneumococcal conjugate vaccine use on invasive pneumococcal disease among adults with and without underlying medical conditions—United States. Clin Infect Dis 2019. Epub August 12, 2019.

If a decision to administer PCV13 is made, it should be administered before PPSV23 (*5*). The recommended intervals between pneumococcal vaccines remain unchanged for adults without an immunocompromising condition, CSF leak, or cochlear implant (≥1 year between pneumococcal vaccines, regardless of the order in which they were received) (*5*). PCV13 and PPSV23 should not be coadministered.

ACIP continues to recommend PCV13 in series with PPSV23 for adults aged ≥19 years (including those aged ≥65 years) with immunocompromising conditions, CSF leaks, or cochlear implants (Table 1) (*2*).

**PPSV23 for adults aged ≥65 years**. ACIP continues to recommend that all adults aged ≥65 years receive 1 dose of PPSV23. A single dose of PPSV23 is recommended for routine use among all adults aged ≥65 years (*1*). PPSV23 contains 12 serotypes in common with PCV13 and an additional 11 serotypes for which there are no indirect effects from PCV13 use in children. The additional 11 serotypes account for 32%–37% of IPD among adults aged ≥65 years (*22*). Adults aged ≥65 years who received ≥1 dose of PPSV23 before age 65 years should receive 1 additional dose of PPSV23 at age ≥65 years (2), at least 5 years after the previous PPSV23 dose (Table 1) (*5*).

## Future Research and Monitoring Priorities

CDC will continue to assess the safety, implementation and the impact of shared clinical decision-making regarding administration of PCV13 to adults aged ≥65 years; the indirect effect of pediatric PCV13 vaccination on disease burden among older adults; and the emergence of nonvaccine serotypes, to inform policy decisions for higher valency conjugate vaccines currently in development. ACIP will continue to review relevant data as they become available and update pneumococcal vaccination policy as appropriate.

Before administering PCV13 or PPSV23, health care providers should consult the relevant package inserts (*21*,*32*) regarding precautions, warnings, and contraindications. Adverse events occurring after administration of any vaccine should be reported to the Vaccine Adverse Event Reporting System (VAERS). Reports can be submitted to VAERS online, by facsimile, or by mail. More information about VAERS is available at https://vaers.hhs.gov/.

**Summary**

**What is already known about this topic?**

In 2014, the Advisory Committee on Immunization Practices (ACIP) recommended 13-valent pneumococcal conjugate vaccine (PCV13) in series with 23-valent polysaccharide vaccine (PPSV23) for all adults aged ≥65 years.

**What is added by this report?**

PCV13 use in children has led to sharp declines in pneumococcal disease among adults and children. Based on a review of accrued evidence ACIP changed the recommendation for PCV13 use in adults.

**What are the implications for public health practice?**

ACIP recommends a routine single dose of PPSV23 for adults aged ≥65 years. Shared clinical decision-making is recommended regarding administration of PCV13 to persons aged ≥65 years who do not have an immunocompromising condition, cerebrospinal fluid leak, or cochlear implant and who have not previously received PCV13. If a decision to administer PCV13 is made, PCV13 should be administered first, followed by PPSV23 at least 1 year later.

## Footnotes

* Recommendations for use of vaccines in children, adolescents, and adults are developed by the Advisory Committee on Immunization Practices (ACIP). ACIP is chartered as a federal advisory committee to provide expert external advice and guidance to the Director of the CDC on use of vaccines and related agents for the control of vaccine-preventable diseases in the civilian population of the United States. Recommendations for use of vaccines in children and adolescents are harmonized to the greatest extent possible with recommendations made by the American Academy of Pediatrics (AAP), the American Academy of Family Physicians (AAFP), the American College of Obstetricians and Gynecologists (ACOG), and the American College of Nurse-Midwives. Recommendations for use of vaccines in adults are harmonized with recommendations of AAFP, ACOG, and the American College of Physicians (ACP). ACIP recommendations approved by the CDC Director become agency guidelines on the date published in the *Morbidity and Mortality Weekly Report*. Additional information is available at https://www.cdc.gov/vaccines/acip.

† Immunocompromising conditions include: chronic renal failure, nephrotic syndrome, immunodeficiency, iatrogenic immunosuppression, generalized malignancy, human immunodeficiency virus, Hodgkin disease, leukemia, lymphoma, multiple myeloma, solid organ transplants, congenital or acquired asplenia, sickle cell disease, or other hemoglobinopathies.

§ Vaccine providers include anyone who provides or administers vaccines: primary care physicians, specialists, physician assistants, nurse practitioners, registered nurses, and pharmacists.

¶ Serotype 6C showed cross-protection from 6A antigen in PCV13 and was grouped with PCV13 serotypes for IPD.

** Immunocompetent defined in discussion as adults without an immunocompromising condition (chronic renal failure, nephrotic syndrome, immunodeficiency, iatrogenic immunosuppression, generalized malignancy, human immunodeficiency virus, Hodgkin disease, leukemia, lymphoma, multiple myeloma, solid organ transplants, congenital or acquired asplenia, sickle cell disease, or other hemoglobinopathies), CSF leak, or cochlear implant.
