# *Erratum*: Vol. 68, No. 46

**DOI:** 10.15585/mmwr.mm685152a3

**Published:** 2020-01-03

In the report “Use of 13-Valent Pneumococcal Conjugate Vaccine and 23-Valent Pneumococcal Polysaccharide Vaccine Among Adults Aged ≥65 Years: Updated Recommendations of the Advisory Committee on Immunization Practices,” on page 1074, the ACIP Pneumococcal Vaccines Work Group should have included **Nancy Bennett, University of Rochester Medical Center**, and **Monica Farley, Veterans Affairs Medical Center and Emory University Department of Medicine**.

